# Healthcare providers' insights on pediatric care quality in Gaza hospitals: integrating evidence-based practices and illness management, health information systems, and referral efficiency

**DOI:** 10.3389/fped.2025.1587984

**Published:** 2025-06-05

**Authors:** Saeed Y. Eleyyan, Bothyna B. ELssyed Etewa, Fatma Al’Haj Ahmad, Abdel Hamid El Bilbeisi

**Affiliations:** ^1^ Department of Pediatric Nursing, School of Nursing, University of Albutana, Rufaa, The Republic of the Sudan; ^2^ Department of Clinical Nutrition, Faculty of Applied Medical Sciences, Al Azhar University of Gaza, Gaza Strip, Palestine; ^3^ Department of Nutrition, School of Medicine and Health Sciences, University of Palestine, Gaza Strip, Palestine

**Keywords:** evidence-based practices, health information systems, healthcare providers, illness, pediatric

## Abstract

**Background:**

The quality of pediatric care in Gaza hospitals is a critical concern, especially given the region's limited resources and ongoing political instability. This study aimed to assess healthcare providers' perspectives on the quality of pediatric care in Gaza's major hospitals, focusing on the integration of evidence-based practices (EBPs) and illness management, health information systems (HIS), and referral efficiency.

**Methods:**

A cross-sectional study was conducted in 2023 at three major pediatric hospitals in the Gaza Strip: Al-Nasr Pediatric Hospital, EL-Dorra Pediatric Hospital, and Al-Rantisi Specialized Pediatric Hospital. A structured interview-based questionnaire was used to collect data from the healthcare providers. Three domains of the World Health Organization (WHO) integrated tool, “Standards for Improving the Quality of Care for Children in Health Facilities” was employed to assess pediatric care quality. Statistical analysis was performed using SPSS version 26.

**Results:**

The study's participants (336 healthcare providers) were predominantly male (59.5%), with an average age of 36.2 ± 8.73 years. Most were nurses (71%), but only a small fraction (0.6%) were pediatric nurses. A significant portion (64.6%) held a bachelor's degree, and a majority (83.3%) had not pursued specialized studies in pediatric care. However, more than half (58.3%) had attended pediatric care training courses, and 23.2% had less than five years of experience. Findings revealed that the integration of EBPs was inconsistent, with only 66.7% of participants reporting proper triage and assessment of emergency cases. Illness management, particularly in areas like malnutrition and anemia, showed significant gaps, with less than 60% adherence to standard practices. The HIS was underdeveloped, with 69.4% of providers reporting accurate medical records, and only 63.7% indicated proper use of data for quality improvement. Referral efficiency was also compromised, with only 65.2% of providers reporting timely referrals.

**Conclusion:**

While some progress has been made in pediatric care quality in Gaza, significant gaps remain in evidence-based practice, illness management, HIS functionality, and referral efficiency. Addressing these challenges requires improved infrastructure, resource allocation, and enhanced training for healthcare providers.

## Introduction

The quality of pediatric care in hospitals is a critical concern for healthcare systems worldwide, and this is particularly true in conflict zones such as the Gaza Strip. In regions with limited resources and ongoing political instability, healthcare providers face numerous challenges in delivering optimal care, especially to vulnerable populations such as children (1). The Gaza Strip, a territory marked by a strained healthcare infrastructure, has been the focus of several studies on healthcare quality (2, 3). To the best of our knowledge, few studies have comprehensively examined pediatric care through the lens of healthcare providers' perspectives, especially in terms of integrating evidence-based practices (EBPs) and managing illnesses effectively, utilizing health information systems (HIS), and ensuring efficient referral processes (4, 5).

EBPs are essential to improving patient outcomes in pediatric care, ensuring that treatment decisions are informed by the latest research and clinical guidelines (6). Yet, in regions with limited access to updated clinical data and resources, the challenge lies in integrating EBPs consistently into daily practice. Illness management is also a major concern, as healthcare providers in Gaza often contend with limited access to medications, medical supplies, and specialized care, which affects their ability to manage complex pediatric cases effectively (7).

The use of actionable HIS plays a critical role in improving healthcare delivery by enabling providers to make informed decisions and track patient outcomes over time. HISs are particularly valuable in resource-constrained settings, where manual record-keeping and a lack of integration across different health services can hinder timely and effective care (8). In the context of Gaza, the functionality of HISs remains underdeveloped, contributing to delays in patient management and increased risks of medical errors. In addition, the referral process, which is a crucial aspect of pediatric care, is often ineffective due to logistical challenges, lack of communication between healthcare centers, and an overburdened healthcare system (9).

Healthcare providers in Gaza face the dual burden of managing pediatric health in an environment marked by scarcity and instability. The continued blockade and military actions have deeply intensified the humanitarian crisis in Gaza, causing critical shortages of vital necessities, including food, safe drinking water, and medical supplies. These conditions have further burdened an already overwhelmed healthcare system (7). The United Nations International Children's Emergency Fund (UNICEF) reports that over one million children in Gaza are in urgent need of life-saving assistance due to the ongoing humanitarian crisis (10). Understanding their perspectives is essential for assessing the gaps in current pediatric care practices and improving the overall quality of care. Previous research has primarily focused on the perspectives of patients and families or on the availability of resources in these settings (11), but there remains a significant gap in examining the comprehensive views of healthcare providers themselves. Their insights on how EBPs and illness management strategies, HIS, and referrals function within the context of Gaza's healthcare infrastructure can offer critical recommendations for improving care quality.

This research aimed to fill this gap by exploring healthcare providers' perspectives on the quality of pediatric care in Gaza hospitals, focusing specifically on the integration of EBPs and the management of pediatric illnesses, the use of HIS, and the efficiency of referral mechanisms. By identifying key barriers and facilitators in these areas, the study will contribute to a deeper understanding of how to enhance pediatric care delivery in resource-limited settings, such as Gaza.

## Materials and methods

### Study design

This research is an observational, descriptive, and analytical cross-sectional study designed to assess healthcare providers' perspectives on the quality of pediatric care in Gaza hospitals, focusing on the integration of EBPs and illness management, HIS, and referral efficiency.

### Study location and period

The current study was conducted in 2023 before Gaza war, in three major governmental pediatric hospitals in the Gaza Strip: Al-Nasr Pediatric Hospital, EL-Dorra Pediatric Hospital, and Al-Rantisi Specialized Pediatric Hospital.

(1) Al-Nasr Pediatric Hospital: Established in 1962, it is the oldest pediatric hospital in Gaza, providing secondary healthcare services for children up to 12 years old. The hospital has 292 staff members and 121 beds, offering emergency, pediatric, intensive care, and neonatal services; (2) EL-Dorra Pediatric Hospital: Opened in 2000, this hospital provides emergency, admission, and specialized services for children. It has 140 staff and 87 beds, with a radiology department managing 1,000–1,800 referrals annually; and (3) EL-Rantisi Specialized Pediatric Hospital: Founded in 2003 and fully operational by 2006, it is a tertiary facility with 287 staff members. The hospital offers 56 beds for children and 30 for adults across 15 specialized departments, including radiology, handling about 7,800 radiology referrals annually (12).

Unfortunately, recent reports suggest that Al-Nasr Pediatric Hospital, El-Dorra Pediatric Hospital, and Al-Rantisi Specialized Pediatric Hospital have either suffered severe damage or are no longer operational as a result of ongoing conflict and airstrikes (13).

### Study population

Inclusion criteria: The study included all healthcare providers, regardless of gender, working in the selected hospitals in the Gaza Strip, including pediatricians, general doctors, pediatric nurses, and general nurses, who were present during the study period were included in the current study.

Exclusion criteria: Healthcare providers employed for less than six months, volunteers, and those who chose not to participate were excluded from the study.

### Sample size and sampling technique

All participants working in the three selected hospitals at the time of data collection and meeting the inclusion criteria were included using a census sampling method. A total of 402 healthcare providers (pediatricians, general doctors, pediatric nurses, and general nurses) were eligible to be included in the current study.

### Data collection

#### Interview-based questionnaire

A structured, pre-tested and validated questionnaire was used to collect data from each participant. The questionnaire had two sections as follows:

1) Characteristics of the study participants:

Participant characteristics, including age, gender, job role, qualifications, specialized pediatric studies, pediatric training courses, years of experience, training duration, etc.

2) Assessment of the quality of care:

Three domains of the WHO integrated tool, “Standards for Improving the Quality of Care for Children in Health Facilities” was used to assess the quality of care provided to children in the Gaza Strip, focusing on EBPs and illness management, HIS, and referral efficiency. EBPs and illness management domain consist of 15-questions; HIS domain consist of 3-questions; and referral efficiency consist of 3-questions that provide more specificity and content for the prioritized areas for quality improvement (14).

### Pilot study

A pilot study was carried out with 20 participants to evaluate the questionnaire and data collection methods. Feedback from the pilot study led to adjustments being made to the questionnaire to improve clarity and accuracy for the main study.

### Data analysis

Statistical analysis was conducted using SPSS version 26. The data analysis process included defining variables, data entry, cleaning, and analysis. Continuous variables were expressed as means ± SD, while categorical variables were presented as percentages. The Chi-square test was applied to assess differences between categorical variables. A *p*-value of less than 0.05 was considered statistically significant.

## Results

### Characteristics of the study participants

This study was conducted in the pediatric departments of three major pediatric hospitals in the Gaza Strip under the Palestinian Ministry of Health: Al-Nasr Pediatric Hospital, EL-Dorra Pediatric Hospital, and Al-Rantisi Pediatric Hospital, which provide care for children up to 12 years old. A total of 402 healthcare providers (pediatricians, general doctors, pediatric nurses, and general nurses) were eligible, with 336 were included in the final analysis, resulting in a 94% response rate. Sixty-six healthcare providers either refused to participate or were absent during the data collection period. Table 1 shows that 59.5% of participants were male, with an average age of 36.20 ± 8.73 years. Most participants (71%) were nurses, and only 0.6% were pediatric nurses. About 64.6% had a bachelor's degree, with statistically significant differences observed between job classification and qualifications by hospital (*P*-value = 0.003). A majority (83.3%) of participants had not completed specialized studies in pediatric care, while 58.3% had attended training courses in pediatric care. Additionally, 23.2% had less than five years of experience, with an average total work experience of 12.6 ± 5.7 years and 9 ± 4.8 years specifically in pediatric care. The average training duration was 10.3 ± 13 weeks. Statistically significant differences were found between special studies in pediatric care and training duration across hospitals (*P*-value = 0.002 and 0.020, respectively).

**Table 1:** Characteristics of the study participants by hospitals.

Variables	Total (*n* = 336)		AL-Nasser pediatric hospital (*n* = 175)		EL-Rantisi pediatric hospital (*n* = 77)		EL-Dora pediatric hospital (*n* = 84)		*P*-value
	100%		52%		23%		25%		
Age (Mean ± SD) (36.20 ± 8.73)									
20–25 years old	32	9.5%	21	12.00%	1	1.30%	10	11.90%	0.058
26–30 years old	73	21.7%	39	22.29%	17	22.08%	17	20.24%	
31–35 years old	76	22.6%	35	20.00%	21	27.27%	20	23.81%	
36–40 years old	70	20.8%	41	23.43%	19	24.68%	10	11.90%	
>40 years old	85	25.3%	39	22.29%	19	24.68%	27	32.14%	
Gender									
Male	200	59.5%	98	56.00%	43	55.84%	59	70.24%	0.058
Female	136	40.5%	77	44.00%	34	44.16%	25	29.76%	
Job classifications									
Pediatricians	44	13.1%	31	17.71%	2	2.60%	11	13.10%	0.003
General Doctors	54	16.1%	32	18.29%	6	7.79%	16	19.05%	
Pediatric Nurses	2	0.6%	1	0.57%	1	1.30%	0	0.00%	
General Nurses	236	70.2%	111	63.43%	68	88.31%	57	67.86%	
Qualification									
Diploma	48	14.3%	25	14.3%	8	10.4%	15	17.9%	0.003
Bachelor	217	64.6%	105	60.0%	58	75.3%	54	64.3%	
Master	47	14.0%	25	14.3%	10	13.0%	12	14.3%	
Ph.D.	24	7.1%	20	11.4%	1	1.3%	3	3.6%	
Special studies in pediatric care									
No	280	83.3%	134	76.6%	71	92.2%	75	89.3%	0.002
Yes	56	16.7%	41	23.4%	6	7.8%	9	10.7%	
Training courses in pediatric care									
No	140	41.7%	70	40.0%	28	36.4%	42	50.0%	0.175
Yes	196	58.3%	105	60.0%	49	63.6%	42	50.0%	
Years of experience (Mean ± SD) (12.6 ± 5.7)									
<5 years	78	23.2%	48	27.4%	12	15.6%	18	21.4%	0330
5–10 years	74	22.0%	37	21.1%	21	27.3%	16	19.0%	
11–15 years	86	25.6%	45	25.7%	21	27.3%	20	23.8%	
>15 years	98	29.2%	45	25.7%	23	29.9%	30	35.7%	
Years of experience in pediatric care (Mean ± SD) (9 ± 4.8)									
<5 years	98	29.2%	55	31.4%	22	28.6%	21	25.0%	0.569
5–10 years	79	23.5%	40	22.9%	21	27.3%	18	21.4%	
>10 years	159	47.3%	80	45.7%	34	44.2%	45	53.6%	
Training duration weeks									
None	129	38.4%	66	37.7%	28	36.4%	35	41.7%	0.020
<5 weeks	54	16.1%	35	20.0%	6	7.8%	13	15.5%	
5–15 weeks	63	18.8%	29	16.6%	24	31.2%	10	11.9%	
16–30 weeks	42	12.5%	16	9.1%	13	16.9%	13	15.5%	
31–45 weeks	18	5.4%	10	5.7%	2	2.6%	6	7.1%	
>45 weeks	30	8.9%	19	10.9%	4	5.2%	7	8.3%	

Continuous variables were expressed as means ± SD, while categorical variables were presented as percentages. The Chi-square test was applied to assess differences between categorical variables. A *p*-value of less than 0.05 was considered statistically significant.

### Evidence-based care and management of illness

Table 2 shows various aspects of pediatric care as per WHO guidelines in the hospitals. For guide (1.1), which addresses triaging and assessing children for emergency signs, 66.7% of participants reported it was done, with a statistically significant difference (*P* = 0.005). Guide (1.2), which involves the assessment and management of sick infants and newborns for serious bacterial infections, was correctly followed by 75.3% of participants, with no significant differences across hospitals (*P* = 0.087). For guides (1.3–1.5), assessing children with cough, diarrhea, and fever, 67%, 66%, and 73.8% of participants reported proper assessment, with statistically significant differences found for all (*P* < 0.05). Guide (1.6) focused on assessing growth, breastfeeding, and nutrition, while guide (1.7) addressed the risk for acute malnutrition and anemia. These were done well by 58.3% and 50.3% of participants, respectively, with significant differences between hospitals (*P* = 0.034 and 0.001).

**Table 2:** Every child receives evidence-based care and management of illness according to WHO guidelines by hospitals.

Variables	Total (*n* = 336)		AL-Nasser pediatric hospital (*n* = 175)		EL-Rantisi pediatric hospital (*n* = 77)		EL-Dora pediatric hospital (*n* = 84)		*P*-value
	100%		52%		23%		25%		
1.1) All children are triaged & promptly assessed for emergency and priority signs to determine if requires resuscitation and receive appropriate care									
Yes	224	66.7%	117	66.9%	50	64.9%	57	67.9%	0.005
Doubt	53	15.7%	33	18.9%	13	16.9%	7	8.4%	
No	59	17.6%	25	14.3%	14	18.2%	20	23.8%	
1.2) All sick infants, especially small newborns, are thoroughly assessed for serious bacterial infection and receive appropriate care									
Yes	253	75.3%	131	74.8%	52	67.6%	70	83.3%	0.087
Doubt	42	12.5%	23	13.1%	14	18.2%	5	6%	
No	41	12.2%	21	12.0%	11	14.3%	9	10.7%	
1.3) All children with cough or difficult breathing are correctly assessed, classified, investigated, receive appropriate care and antibiotics for pneumonia									
Yes	225	76%	131	74.9%	53	68.9%	41	489.8%	0.001
Doubt	43	12.8%	20	11.4%	13	16.9%	10	11.9%	
No	68	20.2%	24	13.7%	11	14.3%	33	39.3%	
1.4) All children with diarrhea are correctly assessed and classified and receive appropriate rehydration and care, including continued feeding									
Yes	222	66.1%	135	77.2%	60	78%	27	32.1%	0.001
Doubt	35	10.4%	21	12%	12	14.3%	3	3.6%	
No	79	23.5%	19	10.9%	6	7.8%	54	64.3%	
1.5) All children with fever are correctly assessed, classified and investigated and receive appropriate care									
Yes	248	73.8%	125	71.4%	55	71.5%	68	51%	0.002
Doubt	36	10.7%	19	10.8%	12	15.6%	5	6%	
No	51	15.2%	31	17.7%	10	13.0%	10	11.9%	
1.6) All infants and young children are assessed for growth, breastfeeding and nutrition, and their carers receive appropriate support and counselling									
Yes	196	58.3%	98	56%	47	61.1%	51	60.7%	0.034
Doubt	33	12.8%	25	14.3%	14	18.2%	4	4.8%	
No	97	28.9%	52	29.7%	16	20.8%	29	34.5%	
1.7) All children at risk for acute malnutrition and anemia are correctly assessed and classified and receive appropriate care									
Yes	169	50.3%	99	56.5%	40	52%	30	35.7%	0.001
Doubt	58	17.3%	29	16.6%	19	24.7%	10	11.9%	
No	109	32.4%	47	26.9%	18	23.4%	44	52.4%	
1.8) All children at risk of tuberculosis (TB) and/or HIV infection are correctly assessed and classified and receive appropriate management									
Yes	00	00	00	00	00	00	00	00	0.001
Doubt	00	00	00	00	00	00	00	00	
No	336	100%	175	100%	77	100%	84	100%	
1.9) All children are assessed and checked for immunization status and receive appropriate vaccinations according to the guidelines of the WHO expanded program on immunization									
Yes	223	66.3%	110	62.9%	50	55%	63	75%	0.039
Doubt	57	17%	31	17.7%	14	18.2%	12	14.3%	
No	56	16.7%	34	19.4%	13	16.9%	9	10.7%	
1.10) All children with chronic conditions receive appropriate care, and they and their families are sufficiently informed about their condition(s) and are supported to optimize their health, development and quality of life									
Yes	200	59.5%	121	69.1%	46	59.8%	33	39.3%	0.001
Doubt	43	12.8%	20	11.5%	17	22.1%	6	7.2%	
No	93	27.7%	34	19.4%	14	18.2%	45	53.6%	
1.11) All children are screened for evidence of maltreatment, including neglect and violence, and receive appropriate care									
Yes	164	48.8%	84	48.0%	33	42.9%	47	55.9%	0.074
Doubt	61	18.2%	30	17.1%	16	20.8%	15	17.8%	
No	111	33.0%	61	34.9%	28	36.4%	22	26.2%	
1.12) All children with surgical conditions are screened for surgical emergencies and injury and receive appropriate surgical care									
Yes	00	00	00	00	00	00	00	00	0.001
Doubt	00	00	00	00	00	00	00	00	
No	336	100%	175	100%	77	100%	84	100%	
1.13) All sick children, especially those who are most seriously ill, are adequately monitored, reassessed periodically and receive supportive care									
Yes	222	66.1%	120	68.5%	45	58.5%	57	67.9%	0.172
Doubt	35	10.5%	18	10.2%	10	13%	7	8.4%	
No	79	23.5%	37	21.1%	22	28.6%	20	23.8%	
1.14) All children receive care with standard precautions to prevent health care-associated infections									
Yes	117	64.6%	113	64.6%	54	70.1%	50	59.5%	0.003
Doubt	48	14.3%	22	12.5%	12	15.6%	14	16.7%	
No	71	21.1%	40	22.9%	11	14.3%	20	23.8%	
1.15) All children are protected from unnecessary or harmful practices during their care									
Yes	219	65.1%	109	62.3%	56	72.7%	54	64.3%	0.001
Doubt	44	13.1%	29	16.6%	12	15.6%	3	3.6%	
No	73	21.7%	37	21.1%	9	11.7%	27	32.1%	
STANDARD (I) Every child receives evidence-based care and management of illness according to WHO guidelines									
Yes	224	66.7%	118	67.4%	50	64.9%	56	66.7%	0.005
Doubt	35	10.4%	21	12.0%	4	5.2%	10	11.9%	
No	77	22.9%	36	20.6%	23	29.9%	18	21.4%	

Categorical variables were presented as percentages. The Chi-square test was applied to assess differences between categorical variables. A *p*-value of less than 0.05 was considered statistically significant.

Guide (1.8), concerning tuberculosis (TB) and HIV assessment, was not applicable in Gaza's pediatric hospitals, as reported by 100% of participants. For guides (1.9 and 1.10), which involve immunization status and care for children with chronic conditions, 62.3% and 59.5% of participants reported proper management, with significant differences by hospitals (*P* = 0.039 and 0.001). Guides (1.14 and 1.15) related to evidence-based care and managing healthcare-associated infections were followed by 64.6% and 65.1% of participants, respectively, with significant differences found (*P* = 0.003 and 0.001). Lastly, guide (1.12), related to screening children with surgical conditions, was not followed by 100% of participants, as Gaza's pediatric hospitals are not equipped for surgical interventions. For stander I, 66.7% of healthcare providers reported adherence to evidence-based care and illness management, with significant differences across hospitals (*P* = 0.005).

### Health information system

Table 3 indicates that, according to the study participants, guides (2.1 and 2.2) were followed to ensure that every child has a complete, accurate, and up-to-date medical record accessible throughout their care, including discharge and follow-up, and that each health facility has a functional system for data collection, analysis, and performance monitoring. The percentages for these guides were 69.4% and 63.1%, respectively. Statistically significant differences were found across hospitals for both guides (*P* = 0.001). For Standard II, which focuses on the collection, analysis, and use of data to improve children's care, 63.7% of healthcare providers reported adherence to these practices, with statistically significant differences across hospitals (*P* = 0.002).

**Table 3:** The health information system ensures the collection, analysis and use of data to ensure early, appropriate action to improve the care of every child by hospitals.

Variables	Total (*n* = 336)		AL-Nasser pediatric hospital (*n* = 175)		EL-Rantisi pediatric hospital (*n* = 77)		EL-Dora pediatric hospital (*n* = 84)		*P*-value
	100%		52%		23%		25%		
2.1) Every child has a complete, accurate, standardized, up-to-date medical record, which is accessible throughout their care, on discharge and follow-up									
Yes	233	69.4%	113	64.5%	52	67.6%	68	81.9%	0.001
Doubt	39	11.6%	17	9.8%	12	15.6%	10	11.9%	
No	64	19.0%	45	25.7%	13	16.9%	6	7.1%	
2.2) Every health facility has a functional mechanism for data collection, analysis and use for monitoring performance and quality improvement									
Yes	212	63.1%	101	57.7%	40	53.3%	70	83.4%	0.001
Doubt	57	17.0%	29	16.6%	20	26.0%	8	9.6%	
No	67	19.9%	45	25.7%	16	20.8%	6	7.1%	
2.3) Every health facility has a mechanism for collecting, analyzing and providing feedback on the services provided and the perception of children and their families on the care received									
Yes	189	56.3%	99	56.6%	37	48.1%	53	63.1%	0.061
Doubt	65	19.4%	32	18.3%	17	22.1%	16	19.1%	
No	82	24.4%	44	25.1%	23	29.9%	15	17.9%	
STANDARD (II) The health information system ensures the collection, analysis and use of data to ensure early, appropriate action to improve the care of every child									
Yes	214	63.7%	106	60.6%	60	78.0%	48	57.1%	0.002
Doubt	39	11.6%	18	10.3%	8	10.4%	13	15.5%	
No	83	24.7%	51	29.1%	9	11.7%	23	27.4%	

Categorical variables were presented as percentages. The Chi-square test was applied to assess differences between categorical variables. A *p*-value of less than 0.05 was considered statistically significant.

### Referral efficiency

Table 4 shows that, according to the study participants, guides (3.1 and 3.3) were followed as per their knowledge. Specifically, 68.2% of participants reported that every child requiring a referral received appropriate pre-referral care and that referral decisions were made without delay. Additionally, 64.6% stated that for children referred within or among health facilities, appropriate information exchange and feedback occurred. Statistically significant differences were found across hospitals for guides (3.1 and 3.3) with *P*-values of 0.035 and 0.026, respectively. For Standard III, which ensures that children with conditions beyond available resources receive appropriate, timely referrals with continuous care, 65.2% of healthcare providers reported timely actions according to participants' knowledge. No statistically significant difference was observed across hospitals (*P* = 0.208).

**Table 4:** Every child with a condition that cannot be effectively managed with available resources receives an appropriate and timely referral, ensuring seamless continuity of care between hospitals.

Variables	Total (*n* = 336)		AL-Nasser pediatric hospital (*n* = 175)		EL-Rantisi pediatric hospital (*n* = 77)		EL-Dora pediatric hospital (*n* = 84)		*P*-value
	100%		52%		23%		25%		
3.1) Every child who requires referral receives appropriate pre-referral care, and the decision to refer is made without delay									
Yes	229	68.2%	115	65.7%	49	63.7%	65	77.4%	0.035
Doubt	43	11.8%	24	13.7%	11	15.6%	7	8.4%	
No	64	19.0%	36	20.6%	16	20.8%	12	14.3%	
3.2) Every child who requires referral receives seamless, coordinated care and referral according to a plan that ensures timeliness									
Yes	214	63.7%	113	64.6%	42	56.6%	59	60.3%	0.289
Doubt	38	11.3%	20	11.4%	13	16.9%	5	6.0%	
No	84	25.0%	42	24.0%	22	28.6%	20	23.8%	
3.3) Every child referred within or among health facilities, there is appropriate information exchange and feedback to relevant health care staff									
Yes	217	64.6%	123	70.3%	49	63.7%	45	53.6%	0.026
Doubt	36	10.7%	11	6.3%	14	18.2%	11	13.1%	
No	81	24.1%	39	22.3%	14	18.2%	28	33.3%	
STANDARD (III) Every child with condition(s) that cannot be managed effectively with the available resources receives appropriate, timely referral, with seamless continuity of care									
Yes	219	65.2%	116	66.3%	54	70.1%	49	58.3%	0.208
Doubt	28	8.4%	13	7.5%	5	6.5%	10	12.0%	
No	89	26.5%	46	26.3%	18	23.4%	25	29.8%	

Categorical variables were presented as percentages. The Chi-square test was applied to assess differences between categorical variables. A *p*-value of less than 0.05 was considered statistically significant.

## Discussion

The quality of pediatric care in Gaza hospitals, as perceived by healthcare providers, is a complex issue influenced by several factors, including EBPs and illness management, HIS, and referral efficiency. The findings of this study offer valuable insights into the current state of pediatric care in Gaza's hospitals, focusing on the integration of these key areas, and comparing them with international standards and recent studies.

Actually, the ongoing war in Gaza has severely disrupted pediatric healthcare, with the destruction of major hospitals leading to a critical decline in services for children. The United Nations reports that the healthcare system is nearing collapse, with only a few hospitals partially functioning. As a result, child mortality has increased, with many dying from preventable conditions due to lack of access to medical care. This underscores the urgent need to address the escalating healthcare crisis and its severe impact on children (15).

### Integration of evidence-based practices

The integration of EBPs into pediatric care is essential for improving patient outcomes. According to Khraisat et al. (16), EBPs ensure that clinical decisions are made based on the latest research, which is critical in pediatric care, where treatment protocols evolve rapidly. In Gaza, the study revealed that 66.7% of participants indicated that children with emergency conditions were triaged and assessed promptly, in line with EBPs, with statistically significant differences across hospitals. While this is a positive finding, it highlights a gap, as 33.3% of participants did not report proper triage and assessment practices. This result mirrors challenges identified in other conflict zones, where resources are limited, and healthcare providers often struggle to integrate updated evidence into daily practices due to lack of access to current guidelines and training (17, 18).

In the case of managing serious bacterial infections in newborns and infants, 75.3% of participants reported appropriate management, but this result was not statistically significant across hospitals. The ability to manage such infections is a critical aspect of pediatric care, and despite the overall positive results, gaps remain in some hospitals. These findings align with previous studies (19, 20), they discussed the impact of underdeveloped healthcare infrastructure and lack of continuous professional development on the quality of care in conflict settings.

### Illness management and resource constraints

Illness management is a cornerstone of pediatric care, particularly in resource-constrained settings like Gaza. The study revealed significant gaps in managing conditions such as malnutrition and anemia, where only 50.3% of healthcare providers effectively assessed the risk for these conditions. Similarly, only 58.3% of participants adequately assessed growth, breastfeeding, and nutrition. The poor performance in these areas may be due to a combination of factors, including limited training in pediatric nutrition and a lack of specialized resources. This finding is consistent with Hamshari et al. (7), who noted that healthcare providers in Gaza face significant challenges in managing pediatric illnesses due to resource scarcity, including shortages in essential medications and medical equipment. The current study revealed that only 50.3% of healthcare providers in Gaza screen for malnutrition, posing a risk to children's health in the face of food insecurity and poverty. Community Health Workers (CHWs) could address this gap, as demonstrated in Ethiopia and Somali refugee camps, where the use of Mid-Upper Arm Circumference (MUAC) tapes by CHWs led to a 34% increase in detection rates. This affordable and sustainable approach aligns well with Gaza's limited resources and existing healthcare infrastructure (21).

The findings related to immunization (62.3%) and care for children with chronic conditions (59.5%) also suggest a gap in implementing standard practices in pediatric care. Immunization rates in Gaza may be impacted by logistical issues such as access to vaccines, transportation difficulties, and supply chain disruptions, which are common in conflict zones (22). These challenges underscore the need for better integration of resources and improved healthcare infrastructure to support effective illness management.

### Health information systems

HIS play a critical role in improving the quality of care by providing timely access to patient data, enabling healthcare providers to make informed decisions. In this study, 69.4% of participants indicated that children had complete and accurate medical records, with significant differences across hospitals. This finding highlights some progress in record-keeping but also suggests that many children may still not benefit from a fully integrated medical record system. The HIS in Gaza's hospitals remains underdeveloped, which is consistent with findings by (23), who observed that manual record-keeping and a lack of integration between healthcare services are common barriers to effective patient management in Gaza.

The functionality of HIS is essential for monitoring patient outcomes and improving care delivery, especially in a resource-limited setting. The study found that 63.7% of participants reported the collection, analysis, and use of health data for quality improvement, with significant differences across hospitals. Although this reflects some progress, HIS in Gaza's hospitals still face many limitations, such as poor integration between hospitals, delays in data processing, and frequent gaps in information sharing. These challenges often lead to delays in decision-making and an increased risk of medical errors (24). Additionally, despite progress, the rate is not higher due to factors such as inadequate training, which limits staff's ability to effectively use data, and technology issues like outdated software or poor infrastructure. Addressing these challenges with better training and technological upgrades could improve data usage across hospitals.

### Referral efficiency

Referral efficiency is another critical aspect of pediatric care, particularly in settings like Gaza, where hospitals often lack the necessary resources to treat complex cases. The study showed that 68.2% of healthcare providers reported that children requiring a referral received appropriate pre-referral care, and 64.6% noted that there was adequate information exchange and feedback during referrals. These findings reflect the importance of timely and effective referrals for managing pediatric conditions that cannot be treated within the hospital. However, the study also highlighted some deficiencies in the referral process, with only 65.2% of healthcare providers reporting timely referrals, and no statistically significant differences across hospitals. This indicates that while there is some level of efficiency in the referral process, logistical challenges, communication barriers, and overburdened healthcare systems continue to hinder optimal care. The issue of referral inefficiency in Gaza's hospitals is consistent with findings of previous studies (25, 26), they noted that the lack of communication between healthcare centers and the overburdened healthcare system leads to delays in treatment and discontinuity of care. Improving referral systems in Gaza requires addressing these systemic challenges, including improving the coordination between hospitals and establishing robust mechanisms for patient information exchange.

This study on pediatric care quality in Gaza hospitals highlights key challenges and opportunities within strained healthcare systems. It emphasizes the importance of integrating EBPs and improving illness management, HIS, and referral efficiency. These findings are relevant not only to Gaza but also to other regions with similar healthcare challenges, such as conflict zones and low-resource settings. By focusing on these areas, the study provides guidance for healthcare providers in resource-constrained environments, aiming for more effective care. The broader implications can inform policy and practice globally, driving improvements in pediatric care, especially in settings facing limited resources and high patient demand.

Additionally, the results of the current study indicated that TB and HIV assessments were not applicable in any of the pediatric hospitals in the Gaza Strip, as reported by all participants. This was primarily attributed to a lack of necessary resources and the low incidence rates of TB and HIV in the Gaza Strip.

Finally, the findings of this study build upon and reinforce our previous work (27), in which we evaluated pediatric care in Gaza using the WHO integrated quality assessment tool. In that earlier study, we identified systemic challenges such as resource limitations, fragmented health service integration, and the urgent need for capacity-building across pediatric hospitals. The current study further confirmed these issues and highlights ongoing gaps in the implementation of evidence-based practices, the functionality of health information systems, and the efficiency of referral processes. Together, these studies emphasize the critical need for targeted interventions to improve the quality of pediatric care in Gaza and other conflict-affected or resource-constrained settings.

### Strength and limitations

The study's strengths include the use of three domains of the standardized WHO integrated tool for the assessment of pediatric care quality in Gaza hospitals, and a large sample size with a 94% response rate. However, its limitations include the cross-sectional design, limiting the ability to assess trends or causal relationships, potential response bias, and limited generalizability to smaller or private healthcare facilities. Additionally, the study primarily focused on internal factors without fully exploring the external influences, such as political instability or external healthcare support.

## Conclusion

The findings of this study emphasize the need for continued improvement in pediatric care in Gaza's hospitals, with a focus on enhancing EBPs and illness management, HIS, and referral efficiency. While there are some positive outcomes in these areas, significant gaps remain, particularly in the integration of updated practices, the management of complex pediatric conditions, the functionality of HIS, and the efficiency of referral processes. These results align with recent studies conducted in conflict settings, where healthcare providers face numerous challenges in delivering high-quality care due to limited resources and infrastructure. Addressing these gaps will require a multifaceted approach, including improved training for healthcare providers, better resource allocation, and enhanced infrastructure to support the delivery of quality pediatric care.

## Data Availability

The raw data supporting the conclusions of this article will be made available by the authors, without undue reservation.
